# Anti-Inflammatory Properties of Menthol and Menthone in *Schistosoma mansoni* Infection

**DOI:** 10.3389/fphar.2016.00170

**Published:** 2016-06-17

**Authors:** Mauricio G. Zaia, Túlio di Orlando Cagnazzo, Karina A. Feitosa, Edson G. Soares, Lúcia H. Faccioli, Silmara M. Allegretti, Ana Afonso, Fernanda de Freitas Anibal

**Affiliations:** ^1^Laboratory of Parasitology, Department of Morphology and Pathology, Universidade Federal de São CarlosSão Carlos, Brazil; ^2^Department of Patology, Faculdade de Medicina de Ribeirão Preto, Universidade de São PauloRibeirão Preto, Brazil; ^3^Departamento de Análises Clínicas, Toxicológicas e Bromatológicas, Faculdade de Ciências Farmacêuticas de Ribeirão Preto, Universidade de São PauloRibeirão Preto, Brazil; ^4^Departamento De Biologia Animal, Instituto de Biologia, Universidade Estadual de CampinasCampinas, Brazil; ^5^Medical Parasitology Unit, Global Health and Tropical Medicine, Instituto de Higiene e Medicina Tropical, Universidade Nova de LisboaLisbon, Portugal; ^6^Bioanalytical, Microfabrication, and Separations Group, Instituto de Química de São Carlos, Universidade de São PauloSão Carlos, Brazil

**Keywords:** *Schistosoma mansoni*, *Mentha piperita* L., immunomodulation, treatment, anti-inflammatory

## Abstract

Schistosomiasis is a parasitic disease caused by several species of trematode worms and it is believed that more than 261 million people are affected worldwide. New drug development has become essential because there is a risk of the parasite becoming resistant to Praziquantel, the only drug available for this infection. This study evaluated parasitological, immunological and histological parameters in mice infected with *Schistosoma mansoni* and treated with an herbal commercial medicine. This drug consists of menthol (30–55%) and menthone (14–32%). A 60 day treatment regimen with the herbal medicine decreased the number of *S. mansoni* eggs in the feces, liver, and intestine and reduced the number of hepatic granulomas. We observed a reduction of 84% in blood eosinophilia and a decrease in the IL-4 and IL-10 blood levels after treatment. Therefore, we propose that schistosomiasis treatment with this herbal medicine for 60 days has an immunomodulatory and anti-inflammatory action in this animal model for schistosomiasis thus contributing to the decrease in physio pathological effects caused by *S. mansoni* infection.

## Introduction

Schistosomiasis is a parasitic disease caused by *Schistosoma* species and it is believed that more than 261 million peoples are affected worldwide, mainly Africa. Urogenital schistosomiasis is caused by *Schistosoma haematobium* and intestinal schistosomiasis can be caused by several species: *S. guineensis, S. intercalatum, S. japonicum, S. mansoni*, and *S. mekongi* (World Health Organization, 2015). Schistosomiasis pathology results from an immune response against *Schistosoma* eggs and granulomatous inflammation around the deposited eggs, stimulated by soluble eggs antigen (SEA), mainly in the liver and intestine tissues (Hatz et al., 1998; Hams et al., 2013) SEA is known to stimulate an immune type 2 response (Th2) leading to the production of IL-4, IL-5, IL-13, and upregulation of immunoglobulin E (IgE) levels and eosinophils (Hams et al., 2013).

The regulation of the chronic phase of schistosomiasis seems to be related to the release of SEAs antigens for the production of different cytokines and the modulation and regulation of the expression of integrins that lead to the recruitment of inflammatory cells to the site of injury induced by the parasite. Some cytokines are more studied and are well-described as being involved in the physio-pathogenesis profile of schistosomiasis. There is a relationship between the Th2 profile response to SEA, many authors have demonstrated that an average of 6–8 weeks after infection by *S. mansoni* an increase on the expression of IL-5 and IL-4 occurs in the serum in response to the presence of eggs in the portal circulation (Pearce and MacDonald, 2002; Swartz et al., 2006). The regulatory cytokines are released in order to contain the process that once chronic, favors hepatic fibrosis. IL-10 cytokine has been implicated as a potent suppressor of effector functions of macrophages, T cells and NK cells (Sadler et al., 2003; Helmy et al., 2009). One possibility is that the IL-10 may interfere and regulate the co-stimulatory molecules B7 antigen presenting cells (APC; Ding et al., 1993), resulting in T cell anergy, this was already shown in a murine schistosomiasis model (Cuison et al., 1995; King et al., 2001). Thus, IL-10 has an important role in regulating the onset of acute disease. According McManus and Loukan (2008), IL10 is of fundamental importance in the generation of the conditions for the host protective homeostatic functions in schistosomiasis (Hoffmann et al., 2000) and the CD4CD25 T cells have been identified as the main source of IL-10 in mice infected by *Schistosoma mansoni* (Hesse et al., 2001). Thus, the regulation of these cytokines down in the chronic phase of the disease favors a less virulent response profile and possibly decreasing the mortality associated with the lesions in this phase of the disease.

Granulomatous inflammation of schistosomiasis depends upon TCD4^+^, macrophages (Parra et al., 1992), eosinophils, and collagen fibers (Pearce and MacDonald, 2002). The collagenous deposition in granulomas leads to a progressive obstruction on blood flow, portal hypertension, varicose veins, and splenomegaly (Ross et al., 2002). Praziquantel (PZQ) has been used for over 40 years for the treatment of this helminthiasis, and is the only recommended drug by the World Health Organization (WHO) for treating *Schistosoma* spp. (Wang et al., 2012). A great concern in the scientific community has been generated after various records showing the ability to select PZQ resistance *in vitro* (Chai, 2013) and *in vivo* (Pinto-Almeida et al., 2015) and also a possible sensitivity lost to PZQ (Ismail et al., 1990; Gryseels et al., 2002).

Medicinal plants have been used for centuries in traditional medicine due to its therapeutic values. Peppermint (*Mentha piperita* L.) oil is one of the most popular and widely used, mainly because of its components, menthol and menthone. This herb has antiviral (Herrmann and Kucera, 1967), antibacterial (Kizil et al., 2010), antifungal (Saharkhiz et al., 2012), and analgesic (Leslie, 1978) activity. The pharmacological activities of *M. piperita* L. includes antiparasitic effects (Naranjo et al., 2006; Dejani et al., 2014). Dejani et al. (2014) showed that the ethanol extract of *Mentha piperita* L. has antiparasitic and immunomodulatory properties for the experimental infection with *S. mansoni*.

However, there are no studies evaluating the effect of menthol and menthone against *S. mansoni* infection. This work was carried out to evaluate the anti-parasitic and immunomodulatory effects of menthol and menthone on a murine *S. mansoni* infection model, from a drug already used for the other purposes in humans.

## Materials and methods

### Animals

Five week-old female Balb/c mice SPF (Specific Pathogens Free) weighing between 18–20 g were obtained from the animal housing of the Centro Multidisciplinar para Investigação Biológica (CEMIB) from Universidade de Campinas, Campinas, Brazil. These animals were maintained under standard laboratory conditions throughout the experiments at the Laboratory of Parasitology, Department of Morphology and Pathology, Universidade Federal de São Carlos-UFSCar, São Carlos, Brazil, with water and food *ad libidum*. The project was approved by the Ethics Committee on animal use from this University, CEA UFSCar n° 034/2013.

### Mice infection with *Schistosoma mansoni*

Mice were infected with ±80 *S. mansoni* cercariae, BH strain, (Belo Horizonte, MG, Brazil) using transcutaneous infection by mouse tail immersion protocol for 2 h, with exposure to light at 28°C, as described by Olivier and Stirewalt (1952). After this, the remaining cercariae were counted by light microscopy for quantification of infectious load that is obtained when we subtract from the original number of cercariae that were unable to enter the mouse epidermis. BH strain is maintained in *Biomphalaria glabrata* snail in the Departamento de Biologia Animal, Instituto de Biologia, Universidade de Campinas, Campinas, Brazil.

### Treatment

Mice were separated into different experimental groups, as represented in Table 1.

**Table 1:** **Experimental design and experimental groups**.

**Groups**	**Mice (*n*)**	**Dose**	**Days**
Negative Control	08	–	–
^*^Positive Control	08	–	–
^*^Mentha 15	08	50 mg/kg/0.2 ml/day	46–60 day after infection
^*^Mentha60	08	50 mg/kg/0.2 ml/day	1–60 day after infection
^*^Praziquantel	08	400 mg/kg/0.2 ml/day	Single dose at 45° day after infection

* *Infected with Schistosoma mansoni*.

An herbal commercial medicine currently indicated for the symptomatic treatment of irritable bowel syndrome, Mentaliv—Apsen Farmacêutica S/A—Registration in the Ministry of Health of Brazil under the number 1.0118.0607) composed of menthol (30–55%) and menthone (14–32%), and prepared from *Mentha piperita* L. leaves^1^, was used. This phytotherapeutical medicine presents itself as a gastro capsule containing 200 mg of the active substances. For treatment, the capsule was solubilized and diluted in PBS (Phosphate-Buffered Saline) for administration by gavage of a 50 mg/kg dose. Tablets of Praziquantel (Tenil^®^Vet—Atral Cipan) were solubilized in 2% Cremophor and administered orally at a single dose of 400 mg/kg (Dejani et al., 2014). Each treatment began 46 days after parasite infection, except for Mentha 60 experimental group, which was initiated on the same day of infection. Treatment of the Mentha 15 experimental group was performed for 15 consecutive days, after infection confirmation using Kato-Katz protocol. Two control groups were included: a negative control (mice not infected and kept untreated) and a positive control (mice infected with *S. mansoni* and left untreated). Food and water were suspended for 1 h before each treatment administration. Each experimental group was composed of eight mice and all experiments were performed in duplicate. In order to perform all the parasitological and immunological analyses mice were euthanized using a CO_2_ chamber.

### Parasitological parameters

Parasite load was calculated on day 61 post parasite infection and worm recovery was made by perfusion of hepatic and portal mesenteric vessels (Duvall and Dewitt, 1967). Eggs were counted using Kato-Katz protocol (Katz and Peixoto, 2000). We used the commercial Kit Helm Test (Bio-Manguinhos, FIOCRUZ) and the number of eggs/gram was determined. Briefly, animals were separated into individual cages and fresh feces were used to perform the technique. Feces were subjected to the screening in nylon and the exact amount of feces was determined by filling a known diameter orifice (provided by the kit) and then placed on a glass slide. Feces already arranged on the slide were covered with cellophane green coverslip impregnated with a solution of malachite green (3% aqueous solution) in order to preserve and whitening of the material. In each experiment, one slide was made per animal and read under light microscopy. The number of eggs was determined by the standard formula Helm Test Kit, Bio-Manguinhos: number of eggs counted × 24 = eggs/gram of feces. On the 45th day after infection, it was determined number of eggs per gram of feces of infected animals to confirm infection. On day 60, after 14 days of treatment, the technique was repeated to evaluate the effect of treatments.

### Extraction of blood and cell counts

On day 61 post parasite infection peripheral blood (PB) samples were obtained through the left brachial vein. Absolute leukocytes counts were measured using a Neubauer chamber with Turk solution for1:20 dilution (Gentian Violet 0.002 g, 3% Acetic acid; Stibbe et al., 1985). PB was collected using EDTA as anticoagulant. Absolute number of different leukocytes was calculated from using differential counts on blood smears stained with Panoptic Rapid (LB, Laborclin). Peritoneal cavity (PC) cells were collected after the injection of 3 ml PBS containing 0.5% sodium citrate. Counts for the total number of leukocytes in PC were performed using a Neubauer chamber and differential counts were obtained from slides prepared using cytospin (Serocito Mod. 2400 Fanen; 1000 rpm/3 min) and stained with Panoptic Rapid (LB, Laborclin).

### Cytokines

Commercially available enzyme-linked immunosorbent assay antibodies were used to measure IL-4, IL-10 in plasma according to manufacturer's instructions (R&D Systems^®^). Each assay was performed in duplicate.

### Histology

Liver and bowel of the mice were removed and immediately fixed in10% formalin on day 61 post parasite infection. Specimens were routinely processed, embedded in paraffin blocks, sectioned into sections of 4 μm and stained with Hematoxylin-Eosin and Gomori for examination under light microscopy. Eggs and granuloma were counted in liver and eggs were counted in bowel. Slides were photographed using a Leica DMRX microscope with camera.

### Statistics analysis

Each experiment was performed twice and the data analysis was done using One-Way ANOVA (*One-Way analysis of variance*) test followed by Tukey, using GraphPad Prism 5. Differences were considered significant when *p* < 0.05. Data will be presented as mean ± SEM.

## Results

### Confirmation of infection

All animals exposed to cercariae had 75 or more cercariae penetrated through their tails, demonstrating that the technique was successful and that this is a very effective method to study the experimental murine model for *S. mansoni* schistosomiasis (data not shown). Kato-Katz was performed to confirm the infection on day 45 post parasite infection. Infection was confirmed in all animals (data not shown).

### Parasitological parameters

On day 60 post parasite infection Kato-Katz was performed. Our results showed a decreased in the egg numbers, of 28.4% in the Mentha 15 experimental group and of 8.4% in the Mentha 60 experimental group, when compared to the positive control group, although there was no decrease in the adult worm count in the hepatic portal system veins in these experimental groups.

### Global and differential counts of cells

**Figure 1:**
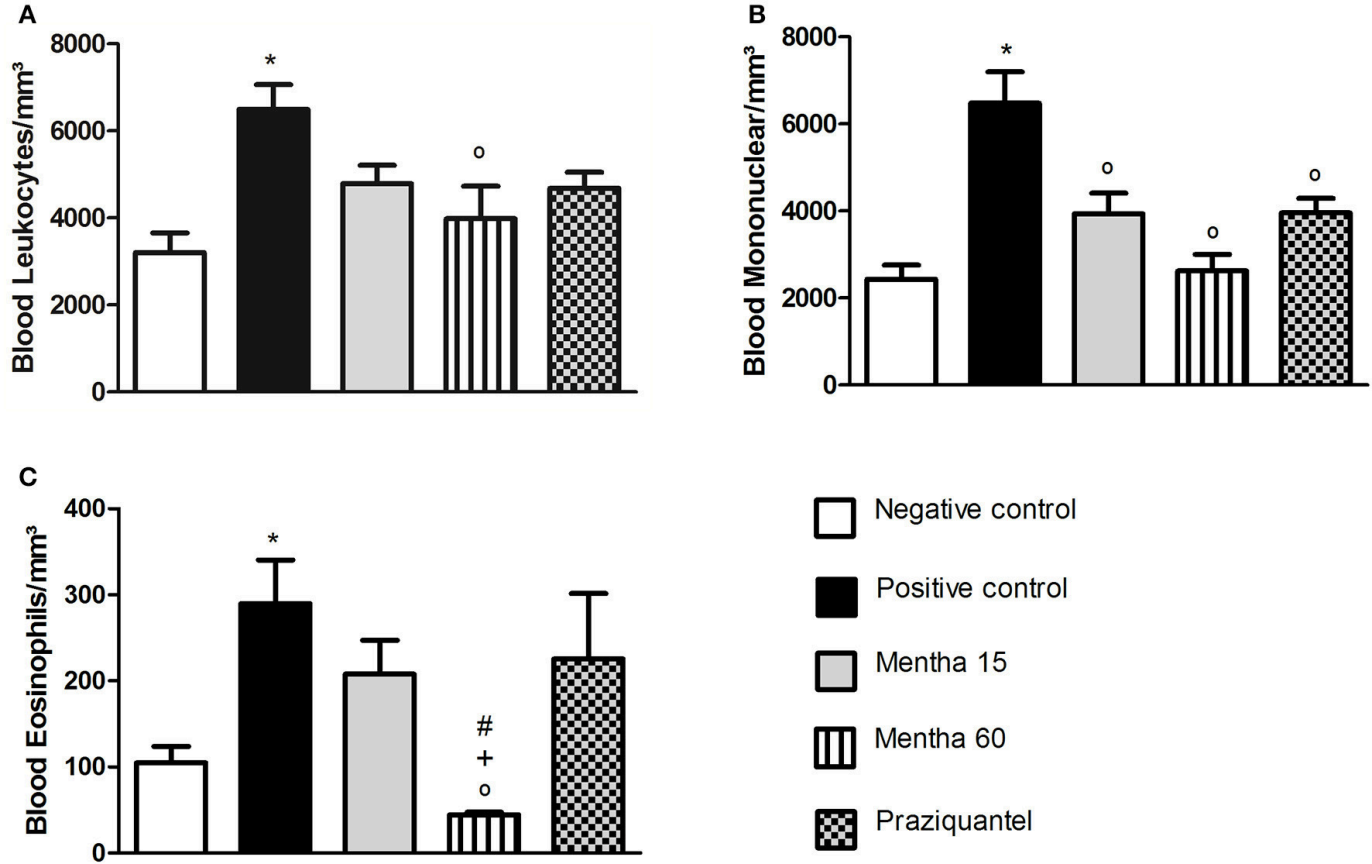
**Global and differential counts of blood leukocytes on day 61 post parasite infection with *S. mansoni***. Blood Leukocytes **(A)**; Blood Mononuclear/mm^3^ **(B)**; Blood Eosinophils/mm^3^ **(C)**. Data is presented as mean ± SEM. Comparison are done between the Negative control (^*^), Positive control (°), Mentha 15 (+), and Praziquantel (#). Differences were considered significant when *p* < 0.05.

Our results showed that both menthol and menthona treatments reduced the number of global leukocytes when compared to the positive control (Figure 1A). The Mentha 15 experimental group presented a decrease of 26.3% while the Mentha 60 experimental group showed a decrease of 38.7% (Figure 1A). A significant decrease (*p* < 0.05) was observed on mononuclear cells. Mentha 15 and Mentha 60 experimental groups presented a reduction of 39.22 and 59.5% respectively, when compared to the positive control group (Figure 1B). Our main outcome was observed in the Mentha 60 experimental group, since this group presented a decrease of 80.9% on blood eosinophilia when compared to the positive control group (Figure 1C).

**Figure 2:**
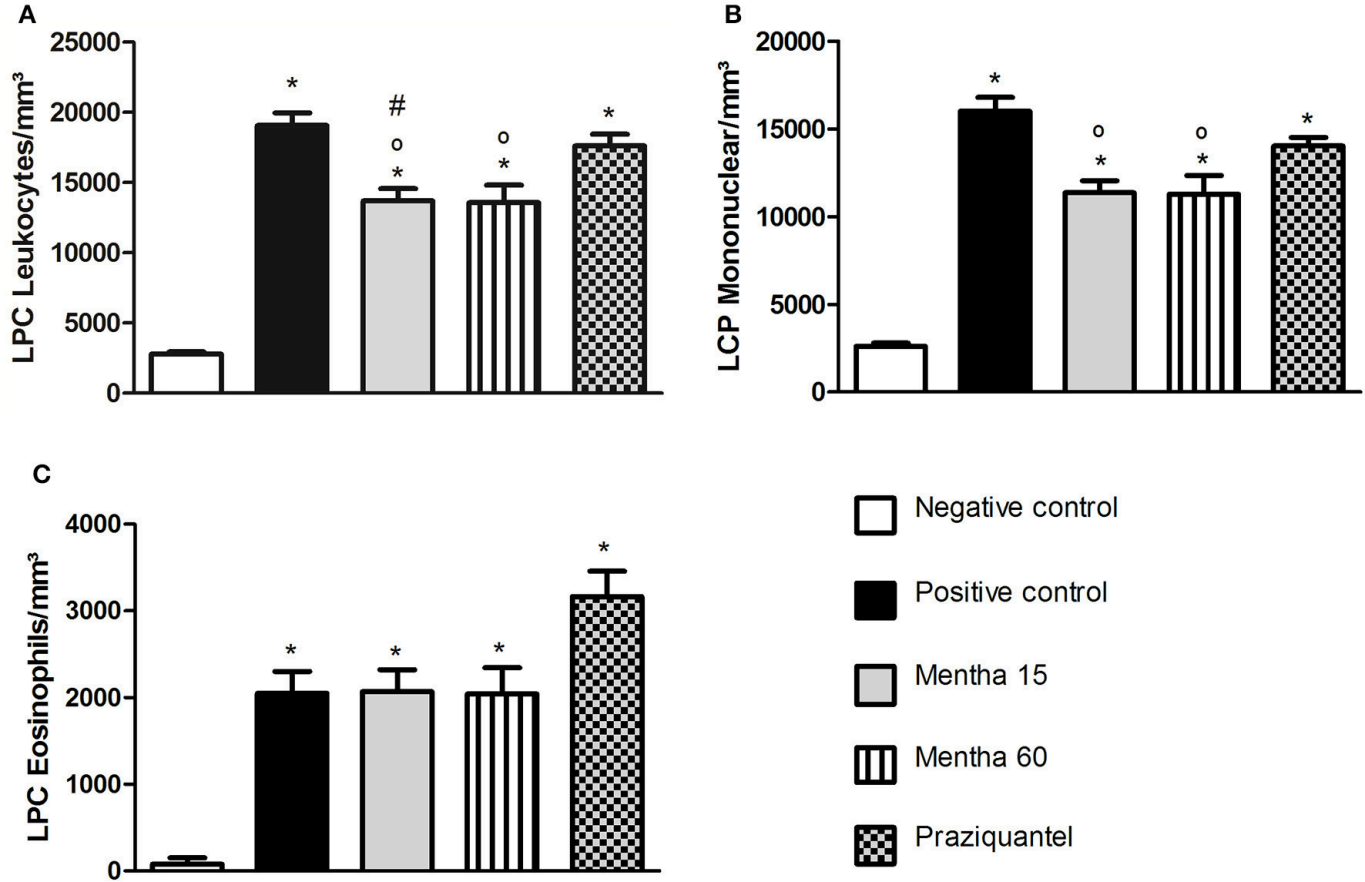
**Global and differential counts of LPC Leukocytes on day 61 post parasite infection with *S. mansoni***. LPC Leukocytes **(A)**; LPC Mononuclear/mm^3^ **(B)**; LPC Eosinophils/mm^3^ **(C)**. Data is presented as mean ± SEM. Comparison are done between the Negative control (^*^), Positive control (°), and Praziquantel (#). Differences were considered significant when *p* < 0.05.

The analysis of global LPC leukocytes (Figure 2A) showed that both treatments were effective on cell reduction. Phytotherapy of Mentha 15 and Mentha 60 experimental groups reduced significantly the number of mononuclear LPC cells by 29.1 and 29.65%, respectively (Figure 2B). The analysis of LPC eosinophils showed no significant change (Figure 2C).

### Cytokines

**Figure 3:**
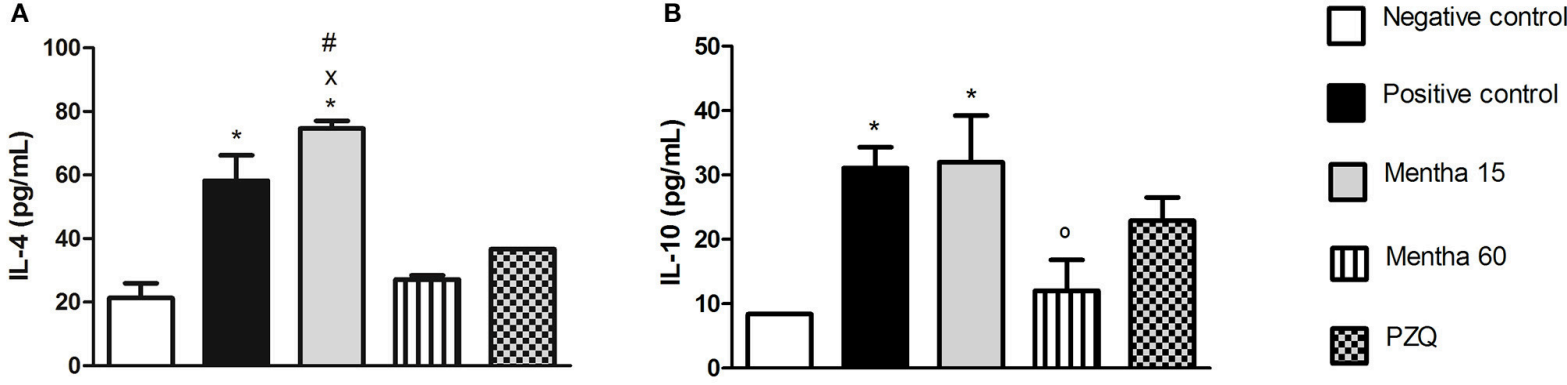
**Cytokines levels on day 61 post parasite infection with *S. mansoni***. IL-4 pg/mL **(A)**; IL-10 pg/mL **(B)**. Data is presented as mean ± SEM. Comparison are done between the Negative control (^*^), Positive control (°), Mentha 60 (x) and Praziquantel (#). Differences were considered significant when *p* < 0.05.

On day 61 post-infection and post-treatment, Mentha 60 experimental group showed a 53.5% decrease in IL-4 levels when compared to the positive control group (Figure 3A). In addition, IL-4 level was lower than the one observed in the Mentha 15 experimental group level (Figure 3A). It was also observed that Mentha 60 experimental group presented a 62% reduction on IL-10 levels when compared to the positive control group (Figure 3B).

### Liver histology

**Figure 4:**
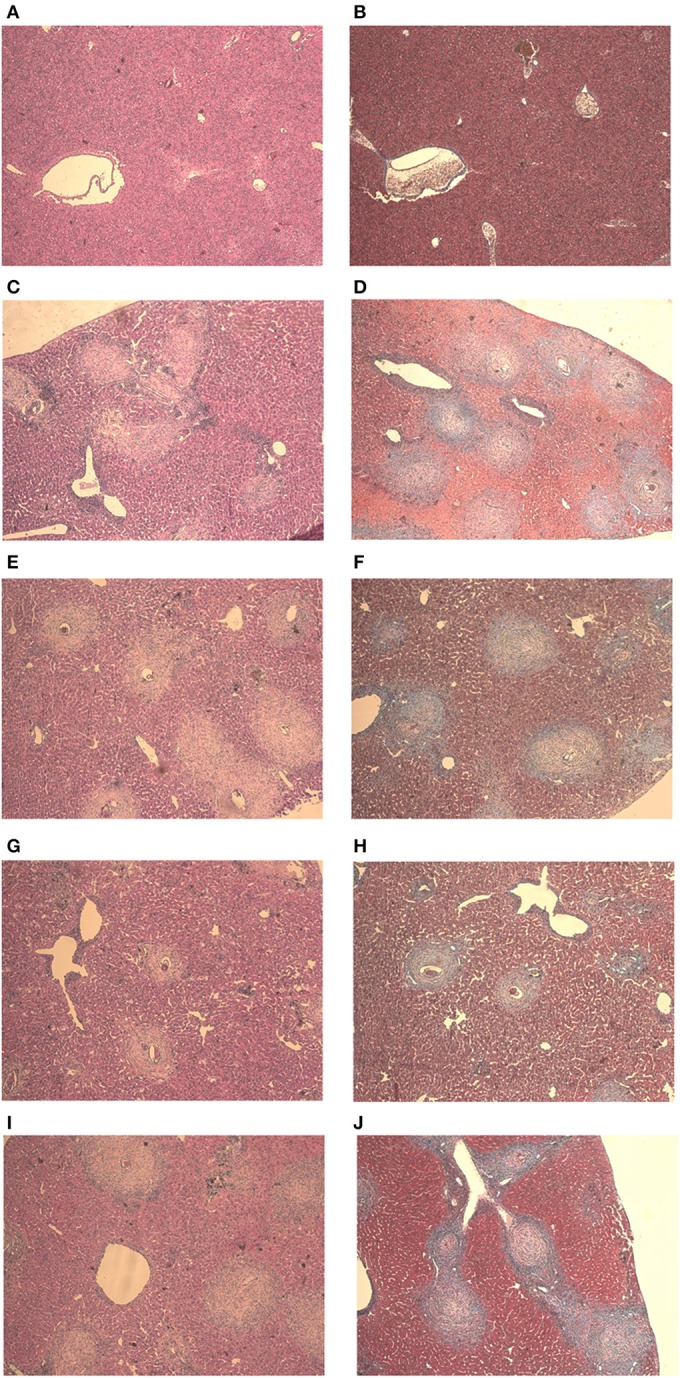
**Liver histology stained with HE and Gomori on day 61 post parasite infection with *S. mansoni***. Negative Control **(A,B)**, Positive Control **(C,D)**, Mentha 15 **(E,F)**, Mentha 60 **(G,H)**, and Praziquantel **(I,J)** are presented with 50x amplification.

Mice from the negative control group showed preserved liver structures and pericanicular and perivascular collagen slender shape. All infected mice showed granulomatous formations around *S. mansoni* eggs, which are formed by lymphocytes, eosinophils, neutrophils and epithelioid cells (Figures 4C,E,G,I). Where there were not eggs deposited, the organs presented its preserved structure (Figures 4A,B).

It was possible to observe the formation of collagenous material in all infected mice organs (Figures 4D,F,H,J). Collagen deposition was observed at a pericanicular and perivascular level but also around eggs (Figures 4D,F,H,J). All groups presented deposition of collagen periovular, indicating the occurrence of a fibrotic scarring process in the liver (Figures 4D,F,H,J). Our results revealed a reduction in egg counts (Table 2) and a decrease in the number of hepatic granulomas on mice from the Mentha 60 experimental group when compared to mice from the positive control group (Table 3). Mentha 15 experimental group presented a reduction in the number of hepatic granulomas (Table 3).

**Table 2:** **Effect of treatment on *S. mansoni* egg number in the liver**.

**Groups**	**Egg/Slide**	**Reduction (%)**
	**Mean ± SEM**	
Positive control	35.2 ± 3.7	–
Negative control	0	–
Mentha 15	35.6 ± 4.3	0
Mentha 60	25.8 ± 2.3	26.7
Praziquantel	15.2 ± 1.7°	56.8

*Eggs were counted in stained slides with HE. One slide per animal was used. Organs were recovered on day 61 post parasite infection. Data is presented as mean ± SEM. Comparison are done between the Positive control and the other groups (°). Differences were considered significant when p < 0.05*.

**Table 3:** **Effect of treatment on number of granulomas in the liver**.

**Groups**	**Granuloma/Slide**	**Reduction (%)**
	**Mean ± SEM**	
Positive control	51 ± 3.8	–
Negative control	0	–
Mentha 15	47.4 ± 3.2	7
Mentha 60	33.8 ± 3.9°	33.7
Praziquantel	32.6 ± 3°	36.1

*Eggs were counted in stained slides with HE. One slide per animal was used. Organs were recovered on day 61 post parasite infection. Data is presented as mean ± SEM. Comparison are done between the Positive control and the other groups (°). Differences were considered significant when p < 0.05*.

### Intestine histology

**Figure 5:**
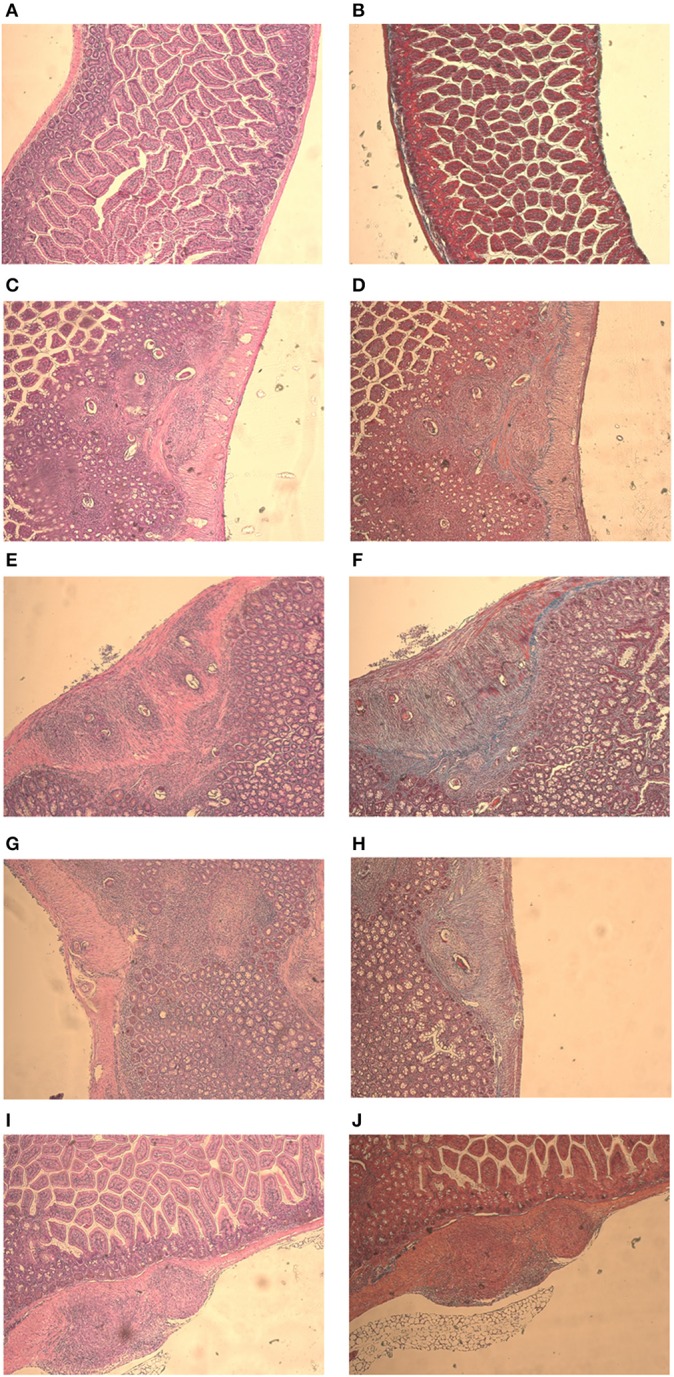
**Intestine histology stained with HE and Gomori on day 61 post parasite infection with *S. mansoni***. Negative Control **(A,B)**, Positive Control **(C,D)**, Mentha 15 **(E,F)**, Mentha 60 **(G,H)**, and Praziquantel **(I,J)** are presented with 50x amplification.

Mice from the negative control group presented intestinal preserved structures (Figures 5A,B). In all infected animals, the presence of eggs in the submucosa and musculature of the intestine with some granulomas was observed (Figures 5C,E,G,I). Histology analysis made clear the presence of collagenous material in all infected mice intestine. The fibrotic process occurred mainly around the eggs, along with the formation of granulomas (Figures 5D,F,H,J). Both phytotherapy treated groups presented an egg count reduction in the intestine, especially Mentha 60 experimental group, which presented a reduction of 41.4% on the total egg count (Table 4).

**Table 4:** **Effect of treatment on number of eggs in the intestine**.

**Groups**	**Egg/Slide**	**Reduction (%)**
	**Mean ± SEM**	
Positive control	123.8 ± 31.2	–
Negative control	0	0
Mentha 15	95.2 ± 13.8	23.1
Mentha 60	72.6 ± 16.9	41.4
Praziquantel	26.4 ± 5.1°	78.7

*Eggs were counted in stained slides with HE. One slide per animal was prepared. Organs were recovered on day 61 post parasite infection. Data is presented as mean ± SEM. Comparison are done between the Positive control and the other groups (°). Differences were considered significant when p < 0.05*.

## Discussion

Reports on *S. mansoni* strains with decreased of sensitivity to PZQ in African countries such as Senegal (Gryseels et al., 2002) and Egypt (Ismail et al., 1990), raise serious concerns in the scientific community, because Praziquantel is the only drug available for schistosomiasis treatment.

Products extracted from plants have been used for many years for disease treatment. Chinese traditional medicine is the most important example of these products efficiency. Complexity, chemical diversity, and biological properties of natural products have led to research and discoveries of new drugs. In Brazil, 100,000 species of plants have been cataloged, but only 8% of them have known chemical components and only 1100 species have been studied for therapeutic use (Castro et al., 2013). Pharmaco-ethnobotany has become a very attractive and with great potential area of research, for new compounds that can be used on infectious diseases treatment.

A wide variety of plants has been tested as agents against various species of *Schistosoma* worldwide for example, artemisinin, a product extracted from the leaves of *Artemisia annua*, which is currently being used in malaria control, can also reduce female worm count and egg release in *S. mansoni* (Lescano et al., 2004). Curcumin, a phenolic compound isolated from the rhizomes of *Curcumin longa*, widely used as spice and food coloring, has been investigated has a potential anti-schistosomal agent (Allam, 2009). Garlic, *Allium sativum* and onion, *Allium cepa*, are both effective in the reducing of the parasitic load of *S. mansoni* in mice by 73 and 66%, respectively (Mantawy et al., 2011).

*Mentha piperita* L., commonly known as peppermint, has components with medicinal activities in all plant parts, including leaves and flowers. *Mentha piperita* L. has a wide range of components, such as menthol (29–48%), menthone (20–31%), menthofuran (6.8%), and methyl acetate (3–10%), representing ~90% of all essential oil, and other pharmacologically active components such as caffeic acid, flavonoids, and tannins (Singh et al., 2011). *Mentha piperita* L. already has their anti-parasitic effects demonstrated. Vidal et al. (2007), has shown *Mentha piperita* L. toxic effects on *Giardia lamblia* trophozoites suggesting *Mentha piperita* L. as a potential phytotherapy agent for giardiasis treatment.

A paper from our group by Dejani et al. (2014) showed that 100 mg/kg *Mentha piperita* L. ethanolic extract has antiparasitic and immunomodulatory properties on *S. mansoni* experimental infection. *Mentha piperita* L. was able to modulate both cells and immune molecules involved in inflammation process, thus suggesting a strong anti-inflammatory effect of this compound in the murine schistosomiasis model. Thus we analyzed the antiparasitic and immunological effects of 50 mg/kg of commercial herbal medicine for human use, consisting of menthol and menthone, extracted from *Mentha piperita* L. leaves, on the murine experimental model of mansonic schistosomiasis. In this study, we used half the dose used by Dejani et al. (2014) because in that work treatment was carried out with menthol and menthone separated. The herbal medicine here tested has defined concentrations of menthol (30–55%) and menthone (14–32%; Mentaliv—Apsen Farmacêutica S/A, Registration in the Ministry of Health of Brazil under number 1.0118.0607) which would allowed a better assessment of their activity against *S. mansoni* infection, since these components can be in a variable concentration in the plant depending on grass climate, soil type, planting ways, and harvesting (Saharkhiz et al., 2012).

Dejani et al. (2014), showed that Balb/c mice infected with *S. mansoni*, LE strain, treated for 49 days with *Mentha piperita* L. ethanolic extract presented a reduction of more than 50% on the total number of eggs in the feces, while observing a reduction of 35.2% on the total number of adult worms recovered by liver perfusion. In this study, animals from both groups treated with 50 mg/kg of the herbal medicine, experimental groups Mentha 15, and Mentha 60, presented a small reduction in the number of eggs in the feces, 28.4 and 8.4% respectively when compared to the positive control group. When we compared the number of adult worms recovered between all treated groups and the positive control group there was no significant difference between those groups. Our results are different from those from Dejani et al. (2014), suggesting that other active components present in *Mentha piperita* L. extract might be acting as active compounds against *S. mansoni*.

In a natural infection, the immunologic response occurs sequentially, such that the Th1 type immune response is down regulated and the Th2 type immune response is up regulated at ~8 weeks after infection (Stavitsky, 2004). Specific cytokines, in particular IL-2, IL-4, IL-5, IL-10, and IFN-^ɤ^ have been implicated in regulation of granulomatous response on schistosomiasis (King et al., 2001). Severe forms of this disease are associated with Th1-type cytokines, whereas Th2-type is correlated with reduction of the pathology and a more benign disease course (Stadecker and Hernandez, 1998). In our study, mice were sacrificed 8 weeks after infection (61 days post parasite infection), the peak release of Th2-type cytokines such as IL-4 and IL-10, was measured in this work.

IL-4 is an important cytokine for the initiation of Th2-type inflammatory response (Silveira-Lemos et al., 2008). Depletion of a Th2-type immune response, particularly IL-4, leads to tissue damage and host mortality due to pro-inflammatory Th1-type response. Th2 response also displays a protective function to the host and minimizes the global burden of host disease (Colley and Secor, 2014). Mentha 15 experimental group showed no significant change in IL-4 levels when compared to the positive control group (Figure 3A). These results corroborated with histological analysis (Figures 4, 5). Treatment was not able to decrease the number of eggs present in the liver (Table 2), showing that this treatment did not prevent egg deposition in the liver and did not alter the inflammatory response profile in this model. We observed a different situation in the Mentha 60 experimental group, which presented a reduction in the IL-4 concentration (Figure 3A).

This result corroborates the histopathological findings, as a decrease number of eggs were observed in the liver and mesenteric tissue, and decreased liver granuloma formation (Table 3). The reduced deposition of eggs in both tissues can have contributed to the decrease in IL-4 secretion in the plasma. Our data suggested that Mentha 60 experimental group has an immunomodulation action, which reduces the Th2-type response, when administered for a long period in this experimental model.

IL-10 may have an anti-inflammatory function by inhibiting the accessory cell function, resulting in decrease of inflammatory cytokines, expression of co-stimulatory molecules, and T lymphocytes regulation (Stadecker and Hernandez, 1998). Low IL-10 production is associated with high risk of fibrosis derivative of *S. mansoni* infection. Thus, IL-10 could reduce the acute pathology of schistosomiasis, regulating the immune response and controlling the chronic morbidity in patients (Silveira-Lemos et al., 2008). Alternatively activated macrophages and IL-10 are part of a regulatory feedback of Th2 response thus limiting the initial granulomatous inflammation (Colley and Secor, 2014). In the Mentha-15 experimental group there was no reduction in the IL-10 anti-inflammatory cytokine expression in this schistosomiasis model when compared to the positive control group (Figure 3B). However, Mentha 60 experimental group showed a significant reduction when compared to the positive control group (62%; Figure 3B), suggesting that a longer treatment period with the herbal medicine comprising menthol and menthone may have an immunomodulatory activity in our experimental murine schistosomiasis model.

Dejani et al. (2014) showed the opposite effect after the 49 days treatment with ethanol extract of *Mentha piperita* L., with significant increase in IL-10 levels when compared with infected and untreated mice. Thus, these further suggests that other components present in the ethanolic extract and absent in the herbal medicine used, can in fact influence the immune response in this experimental murine model of schistosomiasis.

Analysis of blood mononuclear cells revealed that animals treated for 60 days (Mentha 60) showed the lowest level of these cells compared to positive control. Therefore, we suggest that treatment can reduce lymphocytes activation, resulting in low levels of these cells in circulation and low cytokine production. CD4^+^ T cells correspond to 50% of the secreting cell antigens in a granulomatous reaction (King et al., 2001). Animals treated for 15 days (Mentha 15) showed a significant decrease in blood mononuclear cells when compared to positive control group in the same way as the animals treated with PZQ (Figure 1B), but this reduction did not alter the inflammatory response pattern.

Eosinophils have an important protective role in the immune response during *S. mansoni* infection, increasing the number, and activity of these cells, contributing to the role of eosinophils along the infection. In addition, eosinophils are also a major source of cytokines, growth factors, and capable of stimulating the proliferation of fibroblasts and promoting collagen synthesis (Silveira-Lemos et al., 2008). In our study, we observed a decrease of 29.4% in the number of blood eosinophils/mm^3^ in Mentha 15 experimental group when compared to the positive control group (Figure 1C). Mentha 60 experimental group showed a reduction of 84.91% in the blood eosinophils level (Figure 1C). These data corroborates the results from cytokines and histological analysis, further strengthening an immunomodulatory action of menthol and menthone when used for longer treatments. Low IL-4 expression decreases Th2-type inflammatory response, which does not favor eosinophils proliferation and activity especially those stimulated by IL-5 (Swartz et al., 2006).

Thus, our results strongly suggest that this phytotherapeutical medicine (consisting of menthol and menthone) for longer periods has an immunomodulatory and anti-inflammatory action in our murine model of schistosomiasis and it can contribute to the reduction of the pathological effects caused by *S. mansoni* thus to the control of the pathophysiology of the infection in this model.

We suggest that the herbal medicine consisting of menthol (30–55%) and menthone (14–32%), extracted from *Mentha piperita* L. leaves, exhibit an immunomodulatory and antiparasitic effect in the experimental murine model of schistosomiasis. This herbal medicine appears to lead to a decrease in the plasma levels of IL-4 and IL-10, thus indirectly negatively modulating blood eosinophils when used for long periods.

The use of this herbal medicine for longer periods (60 days) appear to reduce the egg number in feces, liver, and intestine and also in reducing the number of hepatic granulomas. Other studies are needed to clarify whether there are other components present in crude extracts and/or essential oils of *Mentha piperita* L. that are responsible for their function in the control of schistosomiasis.

## Author contributions

MZ: Main author of the work. He participated in all analyses and techniques described in the article. He formulated tables, graphs and figures, and participated in the writing of the article. TC: Participation in the parasitological and cellular techniques. He helped in data analysis and participated in discussions related. ES: Realization of assembling and analysis of histological slides in his laboratory, as a collaborator of the work. He participated in the discussion and analysis of histological results. LF: Realization on the immunoassays (ELISA) in his laboratory, as a collaborator of the project. She participated in the discussion and analysis of results this step. SA: Realization the infection of mice with *S. mansoni* in his laboratory. She participated in the analysis and discussion of the results this step. AF: Advisor of project. She actively participated in the work and techniques developed. She assisted in the standardization of methodologies, analysis and discussion of results and in the writing of the article. FA: Main advisor of project. She actively participated in the work and in the techniques developed. She participated of the analysis, discussion and formulation of the results and in the writing of the article.

### Conflict of interest statement

The authors declare that the research was conducted in the absence of any commercial or financial relationships that could be construed as a potential conflict of interest.

## Footnotes

^1^Mentaliv [leaflet]. São Paulo: Apsen FARMACÊUTICA S/A.
